# Inhibition of *Toxoplasma gondii* proliferation by dimethyl itaconate: Evidence from *in vitro* and *in vivo* studies

**DOI:** 10.1371/journal.pntd.0014454

**Published:** 2026-06-18

**Authors:** Jingxian Zhao, Linyan Bao, Huan Chen, Tenglong Zhao, Dong Tang, Yixi Sun, Yuqi Zuo, Jiafu Shang, Yanyan Liu, Xuehui Zhou, Mengru Zhao, Xiaowei Yang, Liwu Zhang, Guangwei Zhao

**Affiliations:** 1 College of Veterinary Medicine, Southwest University, Chongqing, People’s Republic of China; 2 Zhenhe Tongchuang (Chongqing) High-Tech Technology Co., Ltd., Chongqing, People’s Republic of China; NHS Blood and Transplant, UNITED KINGDOM OF GREAT BRITAIN AND NORTHERN IRELAND

## Abstract

*Toxoplasma gondii* (*T. gondii*) is an obligate intracellular parasite capable of infecting more than 350 species, including humans, livestock, and wildlife. However, available clinical drugs for toxoplasmosis not only cause severe adverse effects but also demonstrate reduced therapeutic efficacy due to the emergence of drug-resistant strains, highlighting the urgent need for novel therapeutic interventions. This study aimed to evaluate the activity of dimethyl itaconate (DI) against *T. gondii* both *in vitro* and *in vivo* and to elucidate its underlying mechanism of action. The *in vitro* antiparasitic effects of DI were comprehensively investigated using transmission electron microscopy (TEM), plaque assays, quantitative PCR (qPCR), mitochondrial functional assays, ELISA, and transcriptomic profiling. *In vivo* evaluations were conducted in *T. gondii*-infected mouse models to assess survival rates, parasite loads, histopathological changes, and oxidative stress modulation. The results revealed that DI-treated tachyzoites exhibited marked organelle disruption, loss of membrane integrity, and activation of autophagy. Plaque assays combined with qPCR analysis consistently demonstrated a dose-dependent suppression of *T. gondii* proliferation. Notably, DI induced mitochondrial dysfunction, characterized by reduced mitochondrial membrane potential, ATP depletion, and a concomitant increase in reactive oxygen species (ROS) levels, consistent with the transcriptomic profiling data. This mechanistic evidence suggests that DI exerts its inhibitory effects on *T. gondii* tachyzoites primarily by disrupting the parasite’s energy metabolism pathways. *In vivo*, DI administration increased survival rates, partially alleviated histopathological damage, significantly reduced parasite loads in target organs, and mitigated oxidative stress and inflammatory responses. Overall, DI exhibits promising anti-*T. gondii* activity both *in vitro* and *in vivo*, suggesting its potential as a candidate compound for the treatment of toxoplasmosis.

## Author summary

We investigated whether dimethyl itaconate (DI), a cell-friendly derivative of the natural metabolite itaconate, can combat acute *T. gondii* infections. While itaconate (produced by immune cells during infections) has shown promise against chronic parasitic diseases, its poor cell penetration limits its use. DI solves this problem, but its direct effects on Toxoplasma’s fast-growing “tachyzoite” stage—the primary cause of acute illness—remained unknown. Using lab-grown cells and mouse infection models, we tested how different doses of DI affect parasite growth and host protection. Our results show that this compound not only blocks parasite multiplication but also shields host cells and animals from infection-related damage. By analyzing parasite gene activity, we uncovered key pathways altered by the treatment, revealing how it weakens *T. gondii*. This work provides the first evidence that DI effectively targets acute toxoplasmosis, opening new avenues to repurpose metabolic compounds for fighting infectious diseases.

## Introduction

Toxoplasmosis, a zoonotic parasitic disease with a global distribution, is caused by the intracellular protozoan *Toxoplasma gondii* (*T. gondii*), which can infect nearly all warm-blooded animals [1,2]. Approximately 30% of the world’s population is seropositive for *T. gondii* [3]. The seroprevalence of *T. gondii* in livestock and poultry is higher than that in humans. Specifically, domestic animals such as pigs and sheep exhibit elevated rates [4], whereas chickens, cattle, and dogs show lower seroprevalence rates compared with other livestock species [5]. Furthermore, antibodies against *T. gondii* have been detected in various terrestrial wildlife species and some marine animals, indicating a broader host range than previously recognized [5,6]. The persistence of *T. gondii* imposes significant economic burdens on livestock industries, posing dual threats to animal health and the sustainability of production systems.

The pyrimethamine-sulfadiazine combination remains the first-line therapy for human and veterinary toxoplasmosis [7]. However, its clinical utility is limited by dose-dependent adverse effects, such as hematologic toxicity (e.g., bone marrow suppression) and gastrointestinal disturbances, as well as increasing reports of drug-resistant *T. gondii* strains [8]. Therefore, the discovery of novel anti-Toxoplasma compounds with reduced host cytotoxicity, enhanced parasiticidal activity, and/or synergistic mechanisms remains an urgent priority in parasitology research. Itaconate, an immunoregulatory metabolite produced via immune-responsive gene 1 (*IRG1*)-dependent biosynthesis during tricarboxylic acid (TCA) cycle anaplerosis, competitively inhibits mitochondrial complex II (succinate dehydrogenase, SDH) through structural mimicry of its substrate succinate. This represents a key mechanism in host-directed immunometabolic therapies [9]. This immunometabolite mediates NLRP3 inflammasome suppression by targeted allosteric inhibition of mitochondrial complex II (CII, succinate dehydrogenase), achieved through electrophilic modification of SDHA cysteine residues, thereby coupling TCA cycle remodeling to macrophage immunoregulation [10,11]. Dimethyl itaconate (DI), a membrane-permeable prodrug, recapitulates intracellular itaconate signaling via two mechanisms: covalent modification of KEAP1 cysteine residues (electrophilic activity) and/or allosteric activation of anti-inflammatory receptors. It serves as a chemical biology tool to dissect immunometabolic networks in myeloid cells while bypassing the pharmacokinetic limitations of native itaconate [12]. Recent studies have revealed that DI has distinctive therapeutic potential across multiple domains, including anti-inflammatory, antiviral, antibacterial, and antitumor applications, thereby offering novel strategies for disease prevention and therapeutic intervention [13–19]. Additionally, itaconic acid has been shown to reduce *Schistosoma japonicum* egg burden via the pentose phosphate pathway [20]. In a related study, researchers demonstrated that DI can ameliorate neuroinflammation and cognitive impairment in chronic toxoplasmosis [21]. These findings highlight the potential of developing itaconate-based formulations as immunostimulatory feed additives or antimicrobial/antiparasitic agents. Notably, to date, no studies have systematically evaluated the efficacy of itaconate in controlling *T. gondii* tachyzoite replication in either *in vitro* or *in vivo* models.

In this study, *in vitro* and murine models of *T. gondii* tachyzoite infection were established to systematically evaluate the antiparasitic efficacy of DI through dose–response experiments. Mechanistic insights were gained through differential expression analysis combined with pathway enrichment analysis, identifying key biological processes modulated by DI treatment. These findings provide preclinical evidence supporting the potential of DI as a therapeutic agent for acute toxoplasmosis.

## Materials and methods

### Ethics statement

*In vivo* efficacy was conducted according to the regulations of the Administration of Affairs Concerning Experimental Animals in China. The procedure of animal experiments was approved by the Institutional Animal Care and Use Committee of Southwest University (Approval number: IACUC-20250611–01). Six- to eight-week-old male and female KM mice were purchased from Slack Jingda Laboratory Animal Co., Ltd. (Hunan, China). Before experimentation, all animals were acclimated to the housing conditions for a minimum of a one-week adaptation period, with ad libitum access to food and water.

### Cells and parasites

Baby hamster kidney-21 (BHK-21) cells were obtained from Pricella Biotechnology Co., Ltd. (Wuhan, China). The cells were maintained in high-glucose Dulbecco’s Modified Eagle’s Medium (DMEM, VivaCell, Shanghai, China) supplemented with 10% fetal bovine serum (FBS; VivaCell), 1% penicillin–streptomycin and amphotericin B (Beyotime, Shanghai, China), and cultured at 37°C with 5% CO_2_. Tachyzoites of the *T. gondii* RH strain, maintained in our laboratory, were propagated in BHK-21 cell monolayers using DMEM containing 2% FBS.

### Cytotoxicity assay

The cytotoxicity of DI (Macklin, Shanghai, China) was evaluated in BHK-21 cells using the Cell Counting Kit-8 (CCK-8; Sangon Biotech, Shanghai, China). Cells were seeded in 96-well plates at a density of 2.5 × 10^4^ per well and treated with varying concentrations of DI (0, 45, 60, 73, 75, 90, 105, 120, or 135 μg/mL). After a 24-hour incubation at 37°C in a 5% CO_2_ atmosphere, the drug-containing medium was removed. Subsequently, CCK-8 reagent diluted 1:10 (v/v) in DMEM was added to each well. Following a 40-minute incubation under the same conditions, the optical density at 450 nm was measured using a microplate reader (Infinite 200 PRO, Tecan, Switzerland) to assess cell viability.

### Transmission electron microscopy analysis

For transmission electron microscopy (TEM) analysis, BHK-21 cells were cultured in 6-well plates and inoculated with 6 × 10^6^ *T. gondii* tachyzoites. Meanwhile, the culture medium in the experimental groups was replaced with fresh medium containing 73 μg/mL DI, followed by incubation for 64 hours at 37°C with 5% CO₂. After treatment, infected cells were mechanically detached, pelleted by centrifugation (800 × g, 10 minutes), and washed twice with phosphate-buffered saline (PBS). The cell pellets were then fixed in 2.5% glutaraldehyde in 0.1 M cacodylate buffer (pH 7.4) for 24 hours at 4°C. Macroscopically visible cell-parasite aggregates (approximately 0.5–1 mm in diameter) were selectively collected under stereomicroscopic guidance. Following standard TEM processing—including, postfixation with 1% osmium tetroxide, ethanol dehydration, and epoxy resin embedding, 70-nm ultrathin sections were prepared using an ultramicrotome (Leica EM UC7). Sections stained with uranyl acetate and lead citrate were examined to assess ultrastructural alterations in DI-treated *T. gondii* tachyzoites.

### Plaque assay

BHK-21 cells were cultured in 6-well plates and infected with 6 × 10^6^ *T. gondii* tachyzoites. At the same time, the culture medium in the experimental groups was replaced with different concentrations of DI (57, 65, or 73 μg/mL). After a 64-hour incubation period, the cells were fixed with 4% paraformaldehyde (Biosharp, Beijing, China) at room temperature for 15 minutes and then washed twice with PBS. The cells were subsequently stained with 0.1% crystal violet (Sigma, St. Louis, MO, USA) was performed for 1 hour at room temperature. Following multiple washes with PBS until clear cellular morphology was observed under the microscope, images were captured using phase-contrast microscopy. Quantitative analysis of plaque areas was performed using ImageJ software (v1.53; National Institutes of Health, Bethesda, MD, USA).

### Quantitative PCR

An absolute quantitative PCR (qPCR) approach was employed to evaluate the parasitic load. Genomic DNA was extracted using the FastPure Viral DNA/RNA Mini Kit (Vazyme, Nanjing, China) following the manufacturer’s protocol. Nucleic acid copy numbers in the treatment groups (57, 65, and 73 μg/mL DI) and the untreated group were quantified using pre-established laboratory standard curves [22]. Gene-specific primers targeting the Tg-529 gene were designed as follows: forward, 5′-CACAGAAGGGCCAGAAGT-3′, and reverse, 5′-CATCACCACGAGGAAAGC-3′. All primers were synthesized by BGI Genomics Co., Ltd. (Shenzhen, China). The 20 μL PCR mixture contained 10 μL TB Green Premix Ex Taq (2 × , Takara, Shiga, Japan), 2 μL genomic DNA template (100 ng), 1 μL each of forward and reverse primers (10 µM each), and 5 μL nuclease-free ddH_2_O. qPCR amplification was performed on a QuantStudio 3 system (Applied Biosystems) using TB Green Premix Ex Taq according to the manufacturer’s thermal cycling conditions.

### ATP content assay

The adenosine triphosphate (ATP) content of *T. gondii* was quantified using an enhanced ATP assay kit (Beyotime, Shanghai, China). Experimental groups were established as follows: 2 × 10^6^ *T. gondii* tachyzoites were treated with DI at concentrations of 57, 65, and 73 μg/mL, alongside an untreated control group. After 18 hours of DI exposure, the parasites were collected, lysed on ice with ATP lysis buffer, and centrifuged to pellet cellular debris. The supernatant was carefully aspirated, and the ATP assay working solution was added to the samples according to the manufacturer’s protocol. Chemiluminescence signals were measured using a multimode microplate reader (Infinite 200 PRO, Tecan, Switzerland).

### Mitochondrial membrane potential assay

The mitochondrial membrane potential of *T. gondii* was assessed using a Mitochondrial Membrane Potential Assay Kit (Solarbio, Beijing, China). *T. gondii* tachyzoite cultures (6 × 10^6^ parasites per group) were treated with 57, 65, or 73 μg/mL DI, while an untreated control group was maintained in parallel. After 18 hours of incubation with DI water, the parasites were collected by centrifugation at 300 × g for 5 minutes and washed twice with PBS. Following the manufacturer’s protocol, the harvested tachyzoites were incubated with 1 × JC-1 fluorescent dye for 20 minutes at 37°C in the dark. Finally, the stained parasites were analyzed by flow cytometry (FACSCalibur, BD Biosciences).

### ROS content assay

Intracellular reactive oxygen species (ROS) levels in *T. gondii* were measured using an ROS assay kit (Solarbio, Beijing, China). Briefly, 6 × 10^6^ *T. gondii* tachyzoites were seeded into experimental groups treated with 57, 65, or 73 μg/mL DI, along with an untreated control group. After 18 hours of DI exposure, the parasites were collected by centrifugation and washed twice with PBS. Following the manufacturer’s protocol, the harvested tachyzoites were incubated with 10 μM DCFH-DA fluorescent dye at 37°C for 1 hour in the dark. ROS-mediated fluorescence intensity was then quantified by flow cytometry (FACSCalibur, BD Biosciences).

### Detection of cytokine levels (IL-12, IFN-γ, and TNF-α) via ELISA

BHK-21 cells were cultured in 6-well plates and infected with 6 × 10^6^ *T. gondii* tachyzoites. For the experimental groups, the culture medium was replaced with DI at concentrations of 57, 65, and 73 μg/mL, followed by incubation for 64 hours. The supernatants were then collected, and cytokine levels (IL-12, IFN-γ, and TNF-α) were quantified using specific ELISA kits (Lengton, Shanghai, China) according to the manufacturer’s instructions.

### Transcriptomic sequencing and bioinformatics analysis

A total of 1.6 × 10^7^ fresh tachyzoites were added to each 6-cm cell culture dish. The control group was treated with 2% DMEM without pharmacological additives, while the experimental group received 2% DMEM supplemented with 73 µg/mL DI. After 72 hours of incubation, the cells were mechanically detached and homogenized using a 27G needle. Tachyzoites were then collected, washed twice with PBS, and lysed with TRIzol reagent (Coolaber, Beijing, China).

For transcriptomic profiling, three biological replicates per group were processed. RNA was purified using oligo (dT) bead enrichment, followed by ribosomal RNA depletion to construct stranded cDNA libraries. Raw sequencing data underwent quality control, including adapter trimming, removal of reads containing more than 5% ambiguous bases, and filtration of low-quality sequences. Data integrity was assessed using FastQC (v0.12.1, Babraham Bioinformatics). The reference genome of *T. gondii* was obtained from the NCBI database (version: tgrh88; gene annotation identifier: GCA_013099955.1). High-quality clean reads were aligned to this reference genome using HISAT2 software (Johns Hopkins University, USA). For differential gene expression analysis, stringent thresholds were applied: a false discovery rate (FDR) < 0.05 and an absolute fold change ≥ 2. Gene Ontology (GO) enrichment analysis was performed using the web-based GO resource, with corrected *p*-values < 0.05 indicating statistically significant terms. Additionally, pathway enrichment analysis was conducted using the Kyoto Encyclopedia of Genes and Genomes (KEGG) database.

### Experimental infection design for mice

The experimental design is summarized in Table 1. Briefly, the mice were randomly assigned to five groups, with 15 mice per group. All animals, except those in the normal group, received an intraperitoneal injection of 100 *T. gondii* tachyzoites per mouse. Four hours post-infection, three doses of DI (40, 80, and 120 mg/kg; dissolved in 30% (w/v) PEG300 in sterile distilled water were administered daily via intraperitoneal injection for four consecutive days. The normal group received equivalent volumes of the 30% PEG300 vehicle solution via the same route.

**Table 1:** Grouping, treatment and sampling *in vivo* test.

Group	Treatment	Sampling	
	1 ~ 4 dpi	6 dpi	8 dpi
Normal	0.1 mL 30% PEG 300 + ddH_2_O	Blood samples	Blood samples; Tissue (heart/ liver/spleen/lung/kidney) samples; Peritoneal lavage fluid
Tg	100 *T. gondii* tachyzoites + 0.1 mL 30% PEG 300 + ddH_2_O		
Tg + 40 mg/kg DI	100 *T. gondii* tachyzoites + 0.1 mL 40 mg/kg DI		
Tg + 80 mg/kg DI	100 *T. gondii* tachyzoites + 0.1 mL 80 mg/kg DI		
Tg + 120 mg/kg DI	100 *T. gondii* tachyzoites + 0.1 mL 120 mg/kg DI		

dpi, day post-infection.

Daily health monitoring was conducted using a standardized scoring system, the specific indices of which are presented in Table 2. On 6 dpi, blood samples were collected via retro-orbital puncture from seven mice per group. All remaining animals were humanely euthanized on 8 dpi for the collection of serum and vital organs (heart, liver, spleen, lungs, and kidneys) following established protocols.

**Table 2:** Morbidity symptom scoring scale for *T. gondii*-infected mice.

Ruffled fur	Ascites	Hunched posture	Fecal quality	Activity status
0: Asymptomatic	0: Asymptomatic	0: Asymptomatic	0: Normal	0: Normal
1: Mild	1: Tense ascites	1: Mild	1: Soft stool	1: 25% reduction
2: Severe		2: Severe	2: Watery stool	2: Ambulation only
				3: Non-ambulatory or unresponsiveness
				4: Convulsions or death

### Peritoneal fluid and tissue parasite burden

To assess the *in vivo* inhibitory effect of DI on *T. gondii* proliferation, peritoneal exudate was collected from each experimental group into pre-weighed sterile tubes. Tachyzoite quantification was performed using a hemocytometer-based counting method. Tissue samples (liver, spleen, heart, lungs, and kidneys) were subsequently dissected and snap-frozen in liquid nitrogen for genomic DNA extraction. Parasite burden quantification was conducted via qPCR, with 100 mg tissue aliquots processed according to standardized molecular protocols.

### Histopathological analysis

Following 24 hours of fixation in 4% neutral buffered formalin (NBF), the dissected visceral organs (liver and spleen) underwent standard histoprocessing: dehydration through a graded ethanol series, xylene clearing, and paraffin embedding. The tissue blocks were sectioned into 5-μm slices using a rotary microtome (Leica RM2235) and then stained with hematoxylin and eosin (H&E) according to established histopathological protocols (Macklin, Shanghai, China). Morphological evaluation was performed under a bright-field microscope (Eclipse Ci-L, Nikon, Japan) equipped with a 20 MP digital camera (DS-Fi3), with representative fields captured at 200 × magnification.

### Assays for Alanine aminotransferase (ALT) and aspartate aminotransferase (AST) levels

Hepatic aminotransferase levels were quantified using species-specific commercial kits (Solarbio, China; ALT: BC1555, AST: BC1565), with enzymatic activities expressed as international units per gram of tissue (U/g). Fresh liver samples (100 ± 5 mg wet weight) were cryopreserved at -80°C in RNAlater and subsequently homogenized in ice-cold RIPA buffer (1:10 w/v) using a Precellys 24 TissueLyser homogenizer (3 × 30 s cycles, 6,500 rpm). Lysates were centrifuged at 3,500 × g for 10 minutes at 4°C (Eppendorf 5910 R rotor) to obtain cytosolic fractions. Reaction mixtures were incubated at 37°C for 15 minutes with continuous OD505 monitoring on a microplate reader (Infinite 200 PRO, Tecan, Switzerland). Calibration curves were generated using six-point serial dilutions of standards (0–200 U/L), applying four-parameter logistic regression according to CLSI EP06-A guidelines.

### Assays for Malondialdehyde (MDA) and glutathione (GSH) levels

Hepatic oxidative stress markers (MDA and GSH) were quantified using commercial kits (Jiancheng Bioengineering, Nanjing, China: MDA-A003, GSH-A006) in accordance with the International Federation of Clinical Chemistry (IFCC) guidelines. Fresh liver biopsies (100 ± 2 mg) were homogenized in ice-cold PBS (pH 7.4, 1:9 w/v) containing 0.1% butylated hydroxytoluene using a homogenizer. The homogenate was centrifuged at 2,500 × g for 10 minutes at 4°C (Eppendorf 5910 R rotor) to obtain cytosolic fractions. Total protein concentration was determined using a BCA protein assay kit (Sangon, Shanghai, China). For MDA measurement, 50 μL of the supernatant was reacted with 2-thiobarbituric acid (TBA) at 95°C for 60 minutes under acidic conditions (pH 3.5), producing stable MDA-TBA2 adducts, which were measured at 532 nm with a reference wavelength of 600 nm. GSH levels were analyzed using Ellman’s reagent (5,5’-dithiobis-2-nitrobenzoic acid) in Tris-EDTA buffer (pH 8.0), and thiol groups were quantified at 412 nm after 15 minutes of incubation. Absorbance was recorded using an Infinite 200 PRO microplate reader (Tecan, Switzerland) in 96-well flat-bottom plates (Corning 3599). Analyte concentrations were calculated via four-parameter logistic regression from six-point calibration curves (MDA: 0–50 μM; GSH: 0–1000 μM) and normalized to total protein content (nmol/g protein).

### Detection of serum cytokine levels (TNF-α, IFN-γ, and IL-12) via ELISA

Serum samples were collected from orbital blood of each group on days 6 and 8. The concentrations of the cytokines TNF-α, IFN-γ, and IL-12 in the mouse serum were measured using standardized *in vitro* ELISA methods.

### Statistical analysis

To ensure the reliability and reproducibility of the results, all experiments, including both *in vivo* and *in vitro* studies, were performed with at least three independent biological replicates, and each biological replicate contained 3 technical replicates. All sample sizes and replicate numbers in this study were determined strictly referring to conventional parasitology and molecular biology experimental standards. Three or more biological replicates were adopted to ensure the reliability of results, reduce experimental error, and guarantee sufficient statistical power to detect significant differences using one-way ANOVA followed by multiple comparison tests. All statistical analyses were conducted using GraphPad Prism software (San Diego, CA, USA). Data are presented as the means ± SD. Statistical differences were analyzed using one-way ANOVA or two-way ANOVA followed by Dunnett’s multiple comparisons test. Statistical significance was defined as *p* < 0.05. In all cases, p-values were expressed as *p < 0.05, **p < 0.01 and ***p < 0.001.

## Results

### Cytotoxicity of DI

**Fig 1:**
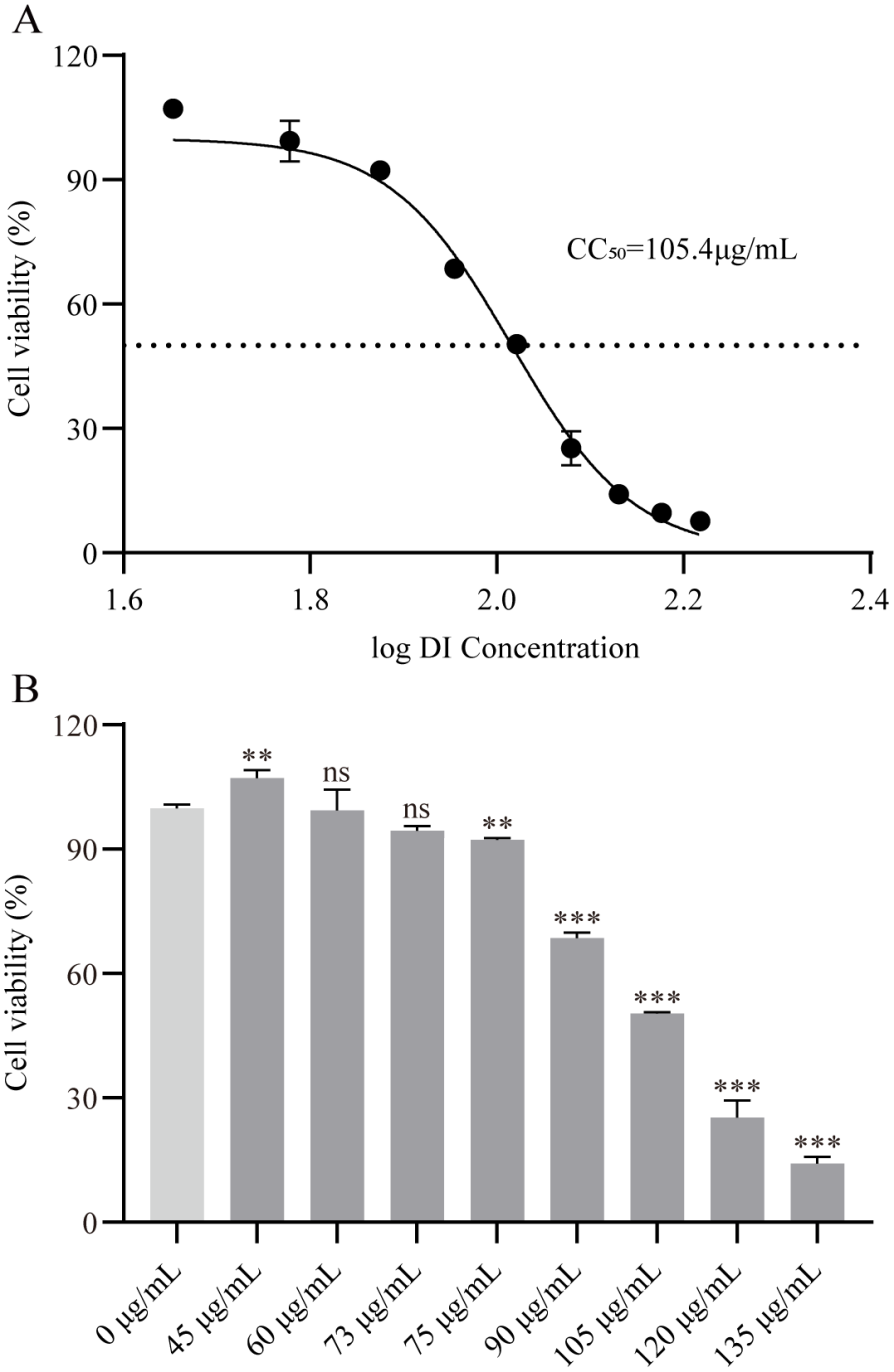
Impact of DI treatment on host cells. Cells were treated with increasing concentrations of DI (0, 45, 60, 73, 75, 90, 105, 120, and 135 µg/mL) for 24 hours. Cell viability was assessed using the CCK-8 assay. A: The 50% cytotoxic concentration (CC_50_) of DI. The CC_50_ value was calculated using nonlinear regression analysis. B: Cytotoxicity of DI on BHK-21 cells. Data are presented as mean ± SD derived from three independent biological replicates, with each biological replicate consisting of three technical replicates. Statistical significance was determined by one-way ANOVA followed by Dunnett’s multiple comparisons test. **p < 0.01, ***p < 0.001, ns = not significant.

The cytotoxicity of DI on BHK-21 cells was evaluated, revealing a 50% cytotoxic concentration (CC_50_) of 105.4 μg/mL (Fig 1A). At concentrations ≤ 73 μg/mL, no statistically significant difference (*p* > 0.05) in BHK-21 cell viability was observed compared to the control groups (Fig 1B). Therefore, 73 μg/mL was adopted as the highest non-toxic concentration for the evaluation of anti-*T. gondii* efficacy. Combined with the inhibitory effects on *T. gondii* observed in preliminary experiments, three graded concentrations (57, 65, and 73 μg/mL) were selected as the low-, medium-, and high-dose groups for subsequent *in vitro* assays.

### Effect of DI on the ultrastructure of *T. gondii* tachyzoites

**Fig 2:**
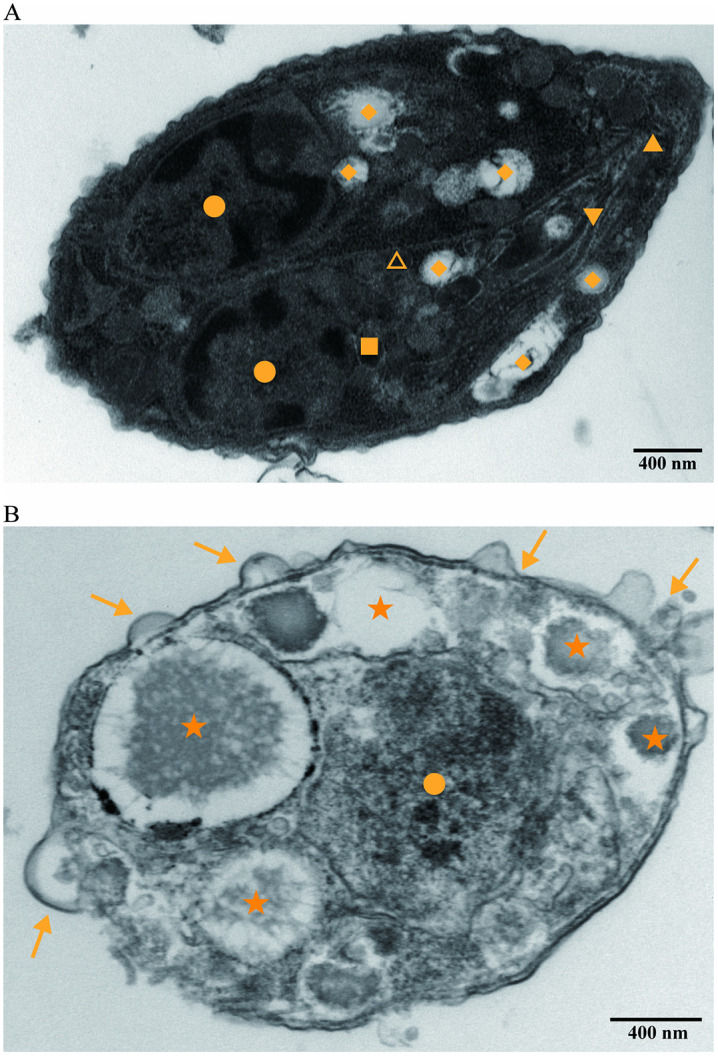
Ultrastructural alterations on tachyzoites of *T. gondii* treated with 73 μg/mL DI for 64 hours. A: Untreated tachyzoite. B: DI-treated tachyzoite. ●, nucleus; ■, mitochondria; ▲, conoid; ▼, microneme; △, dense particles; ◆, starch granules; → , leakage of cytoplasmic contents; ★: autophagic vacuoles.

Transmission electron microscopy (TEM) analysis of DI-treated *T. gondii* tachyzoites revealed significant ultrastructural changes, providing high-resolution evidence of the compounds’ targeted inhibitory effects on parasitic morphology. Control tachyzoites exhibited characteristic intact cellular architecture with clearly defined organelles during active proliferation (Fig 2A). In stark contrast, DI-treated parasites showed severe structural deformation and membrane rupture, accompanied by mitochondrial swelling and loss of organelle definition. Notable pathological features included disintegration of membrane systems and extensive cytoplasmic vacuolization (Fig 2B). These morphological alterations indicate compound-induced mitochondrial dysfunction, abnormal lipid body accumulation, and compromised plasma membrane integrity.

### DI reduced the plaque formation ability of *T. gondii*

**Fig 3:**
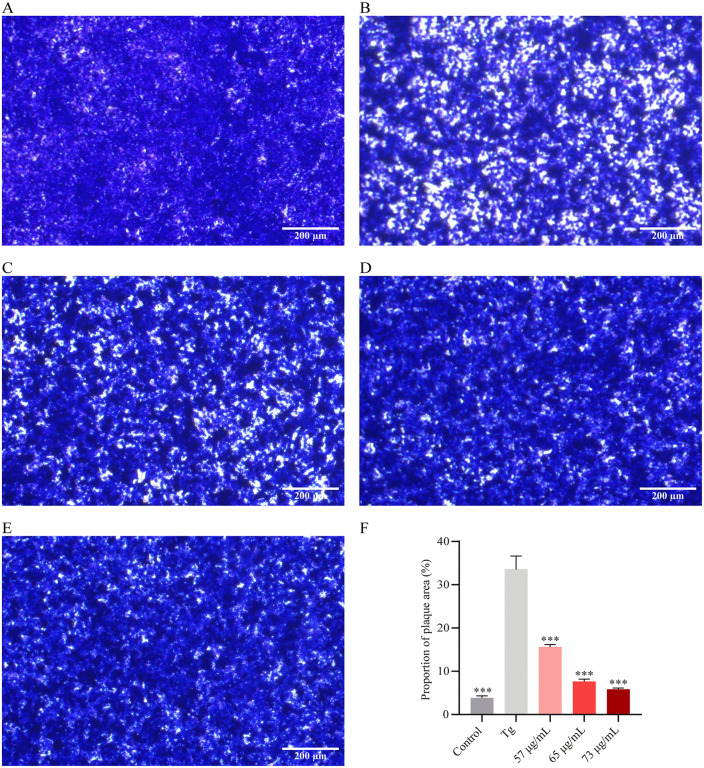
Effects of DI on *T. gondii* tachyzoite plaque formation after 64 hours of DI treatment. A-E: Crystal violet-stained plaque images. A: Uninfected and untreated BHK-21 control. B: *T. gondii*-infected BHK-21 without treatment. C-E: *T. gondii*-infected BHK-21 treated with DI at concentrations of 57, 65, and 73 μg/mL, respectively. F: Quantitative analysis of relative plaque areas. Data are presented as mean ± SD derived from three independent biological replicates, with each biological replicate consisting of three technical replicates. Statistical significance was determined by one-way ANOVA followed by Dunnett’s multiple comparisons test. ***p < 0.001.

The plaque assay quantitatively evaluates *T. gondii* tachyzoite infectivity by measuring the complete lytic cycle progression, including host cell invasion, intracellular replication, and parasite egress. To systematically assess the pharmacological impact of DI on this critical process, comparative plaque formation analyses were performed at three treatment concentrations (57, 65, and 73 μg/mL) versus untreated controls (Fig 3A-3E). The control groups developed well-defined plaques with characteristic radial expansion patterns, whereas the DI-treated groups exhibited markedly reduced plaque formation. Quantitative analysis revealed highly statistically significant suppression across all treatment groups compared to controls (*p* < 0.001, Fig 3F), demonstrating a clear dose-dependent inhibitory effect.

### DI inhibited the proliferation of *T. gondii in vitro*

**Fig 4:**
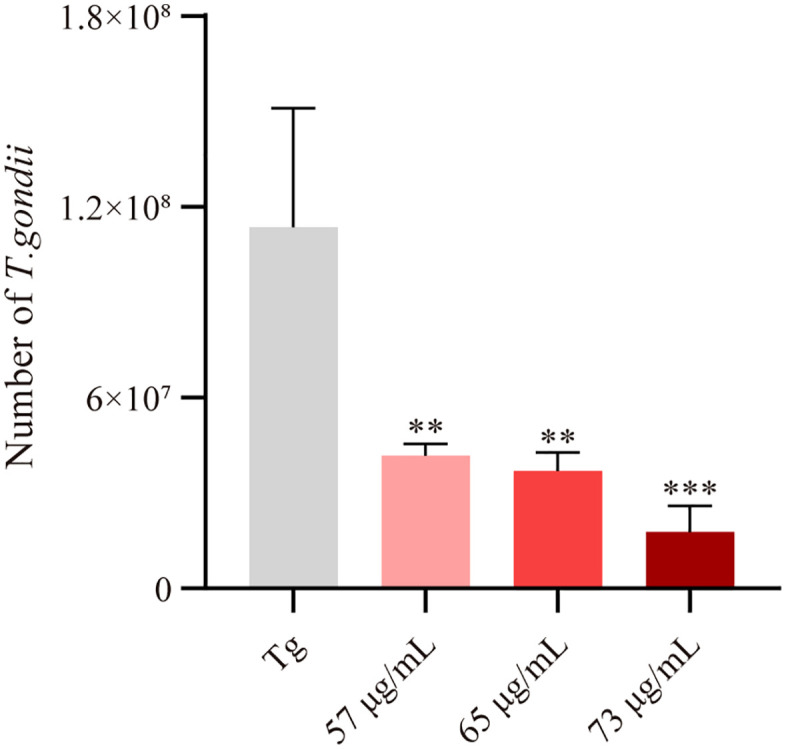
Effects of DI on *T. gondii* tachyzoite proliferation. BHK-21 cells were infected with 5 × 10^6^ *T. gondii* tachyzoites and treated with DI at concentrations of 57, 65, and 73 μg/mL for 48 hours. Tachyzoites were subsequently harvested for analysis. Data are presented as mean ± SD derived from three independent biological replicates, with each biological replicate consisting of three technical replicates. Statistical significance was determined by one-way ANOVA followed by Dunnett’s multiple comparisons test. **p < 0.01, ***p < 0.001, ns = not significant.

Quantitative real-time PCR (qPCR) was employed to quantify parasite nucleic acid levels in both the experimental and control groups, aiming to evaluate the inhibitory effect of DI on *T. gondii* tachyzoites. Under standardized infection conditions, all DI-treated groups exhibited significantly lower parasite DNA loads compared to the infection control group (*p* < 0.01; Fig 4). Moreover, a clear dose-dependent suppression pattern was observed across the tested concentration range.

### DI decreased ATP levels and mitochondrial membrane potential while increasing ROS levels in *T. gondii*

**Fig 5:**
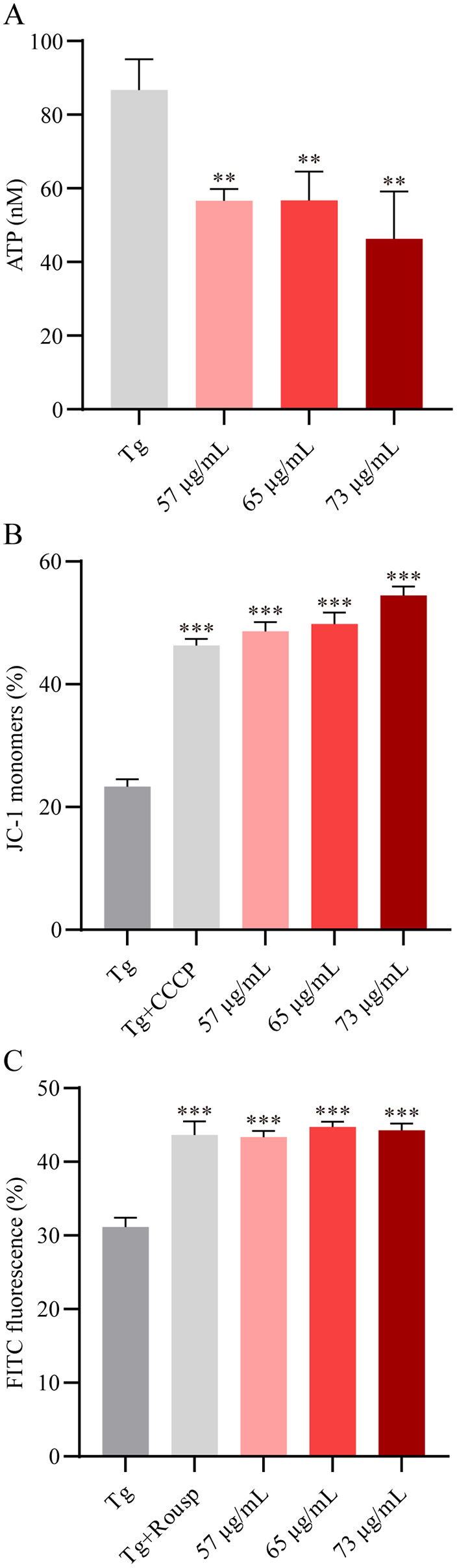
Effects of DI on the mitochondria of *T. gondii* tachyzoites. A: DI-induced alterations in ATP levels of *T. gondii* tachyzoites. Purified tachyzoites were incubated with DMEM or DI (57, 65, and 73 μg/mL) for 18 hours. B: JC-1 monomer fluorescence intensity levels. C: FITC-A fluorescence intensity levels. Data are presented as mean ± SD derived from three independent biological replicates, with each biological replicate consisting of three technical replicates. Statistical significance was determined by one-way ANOVA followed by Dunnett’s multiple comparisons test. **p < 0.01, ***p < 0.001.

**Fig 6:**
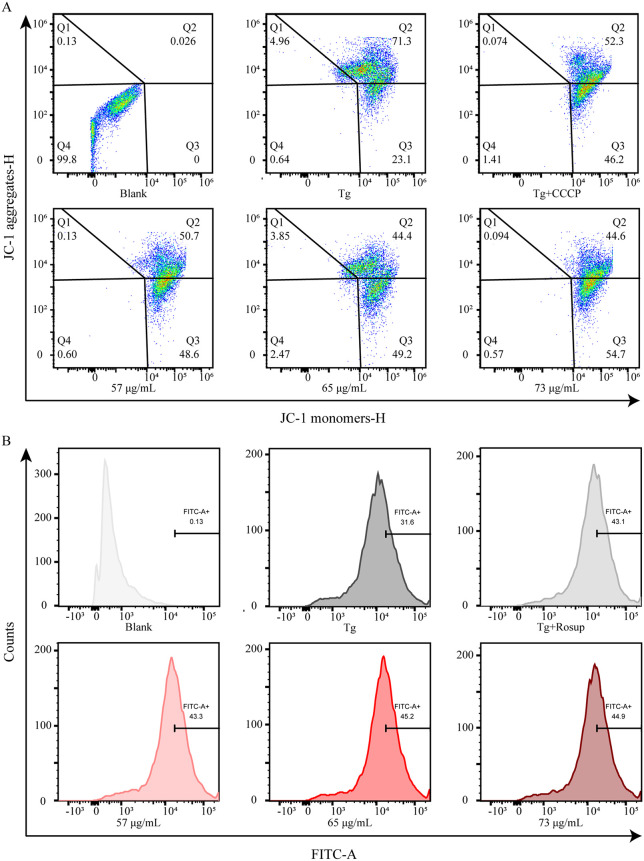
Effects of DI on the mitochondrial membrane potential and ROS levels in *T. gondii* tachyzoites. A Effect of DI on the mitochondrial membrane potential of *T. gondii* tachyzoites. Purified tachyzoites were treated with DMEM, CCCP (positive control), or DI at concentrations of 57, 65, and 73 μg/mL for 18 hours. B Effect of DI on ROS levels in *T. gondii* tachyzoites. Purified tachyzoites were treated with DMEM, Rosup (positive control), or DI at concentrations of 57, 65, and 73 μg/mL for 18 hours. Gating strategy: Flow cytometry data were analyzed using FlowJo software. First, the tachyzoite population was gated based on forward scatter (FSC-A) and side scatter (SSC-A) to exclude debris. For the mitochondrial membrane potential assay, the ratio of red (JC-1 aggregates) to green (JC-1 monomers) fluorescence was calculated within the gated parasite population. For the ROS assay, DCF-positive parasites (indicating ROS production) were quantified within the same gated population.

To elucidate the potential anti-*T. gondii* mechanism of DI, tachyzoites were exposed to varying concentrations of DI. DI treatment induced dose-dependent mitochondrial dysfunction, as evidenced by a significant decrease in ATP production (*p* < 0.01; Fig 5A) and diminished mitochondrial membrane potential (*p* < 0.001; Figs 5B, 6A). These changes were accompanied by a marked increase in reactive oxygen species (ROS) levels (*p* < 0.001; Figs 5C, 6B), as quantified by FITC fluorescence intensity.

### DI reduced the secretion of IFN-γ and IL-12 to alleviate the inflammatory response

**Fig 7:**
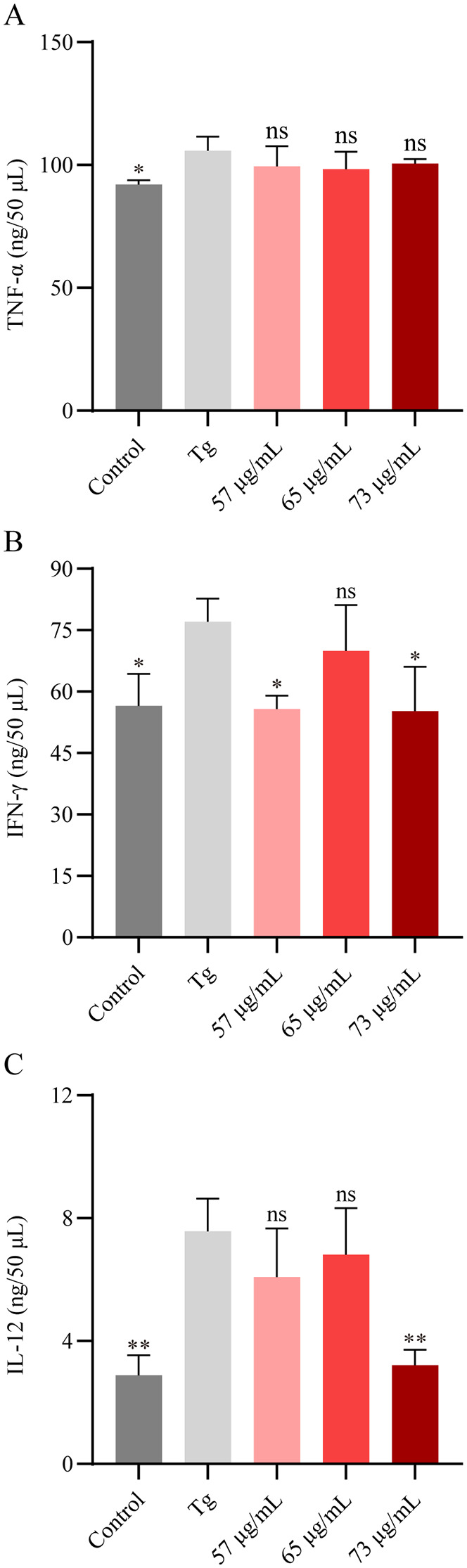
Effects of DI on cytokine production in host cells. BHK-21 cells were infected with 6 × 10^6^ *T. gondii* tachyzoites and treated with DI at concentrations of 57, 65, and 73 μg/mL for 64 hours. Subsequently, cell culture supernatants were collected for cytokine analysis. **A-C:** Levels of TNF-α, IFN-γ, and IL-12 in the supernatants, quantified by ELISA. Data are presented as mean ± SD derived from three independent biological replicates, with each biological replicate consisting of three technical replicates. Statistical significance was determined by one-way ANOVA followed by Dunnett’s multiple comparisons test. *p < 0.05, **p < 0.01, ns, not significant.

To investigate the effect of DI on *T. gondii*-infected BHK-21 cells, we measured the levels of TNF-α, IFN-γ, and IL-12 in cell culture supernatants. DI treatment significantly reduced the *T. gondii*-induced production of IFN-γ (*p* < 0.05; Fig 7B) and IL-12 (*p* < 0.01; Fig 7C) in cells, whereas TNF-α levels did not differ significantly between treated and untreated groups (*p* > 0.05; Fig 7A).

### DI alters the transcriptome of *T. gondii*

**Fig 8:**
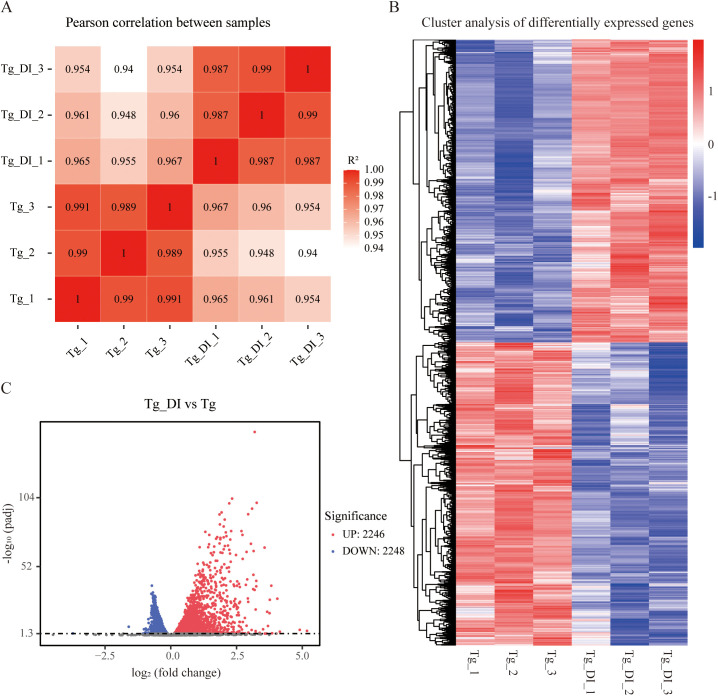
Global transcriptomic profiling and identification of differentially expressed genes (DEGs) in *T. gondii* tachyzoites treated with DI. A: Heatmap displaying Pearson correlation coefficients between biological replicates. B: Unsupervised hierarchical clustering of RNA‑seq data using normalized FPKM values (color scale: blue = low expression, red = high expression). Columns are clustered based on Pearson correlation distance. C: Volcano plot comparing DI-treated and control (DMEM) groups, with the y-axis showing −log_10_ (Q values) and the x-axis representing log₂(fold change) in gene expression.

**Fig 9:**
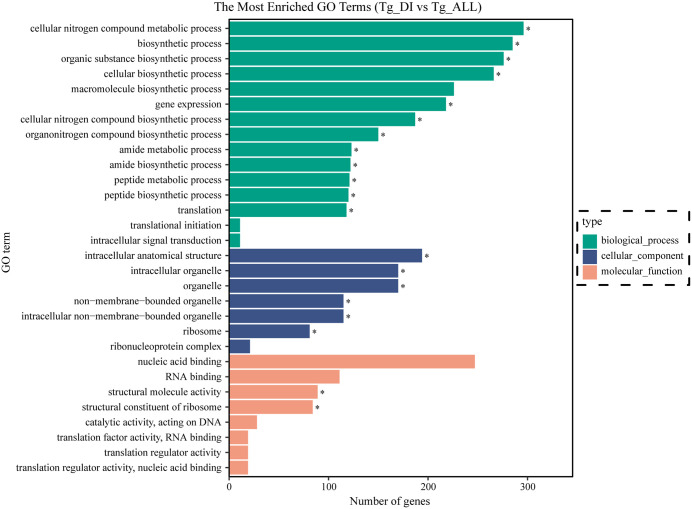
Functional annotation of DEGs by Gene Ontology (GO) enrichment analysis. GO enrichment analysis of DEGs, with the y-axis listing enriched GO terms and the x-axis corresponding to −log₁₀(*p* values) for statistical significance.

**Fig 10:**
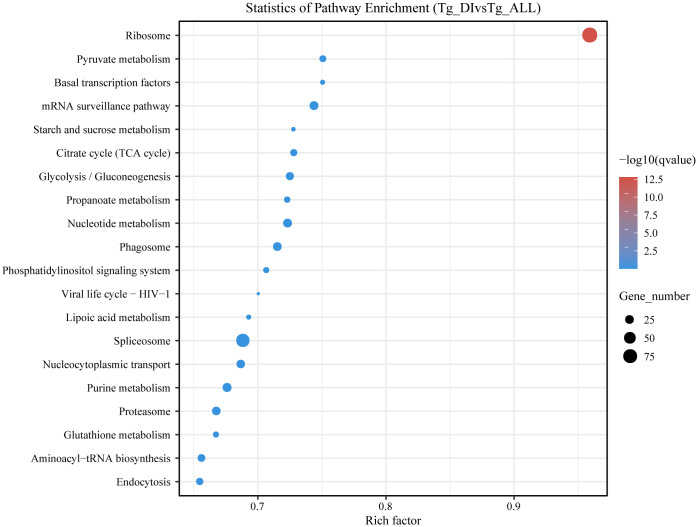
Pathway enrichment analysis of DEGs using the Kyoto Encyclopedia of Genes and Genomes (KEGG) database. KEGG pathway analysis of DEGs. The y-axis displays enriched pathways, and the x-axis shows the enrichment ratio. Dot color intensity reflects the − log₁₀(*p* value), and dot size indicates the number of DEGs per pathway.

To evaluate the effect of DI on *T. gondii* gene expression profiles, we conducted a transcriptomic analysis of parasites treated with 73 μg/mL DI for 72 hours. The experimental design included three biological replicates for both the DI-treated and untreated control groups. Analysis of RNA expression patterns using Pearson correlation coefficient heatmaps revealed distinct transcriptional responses. As shown in Fig 8A, the correlation matrix clearly demonstrated separation between the DI-treated and control groups (Pearson’s r^2^ < 0.97 between experimental conditions vs. r^2^ > 0.98 within groups). Unsupervised hierarchical clustering further confirmed differential gene expression patterns in the treated parasites, with complete segregation from the control parasites (Fig 8B). Differential expression analysis using DESeq2 identified 2,246 significantly upregulated and 2,248 downregulated genes in DI-treated *T. gondii* compared to untreated controls (Fig 8C). To elucidate the biological implications of these transcriptional changes, we performed Gene Ontology (GO) enrichment analysis across three functional categories: biological processes (BP), molecular functions (MF), and cellular components (CC). The most significantly enriched terms included “cellular nitrogen compound metabolic process” (BP), “intracellular organelle organization” (CC), and “oxidoreductase activity” (MF) (Fig 9). Kyoto Encyclopedia of Genes and Genomes (KEGG) pathway analysis revealed DI-mediated modulation of key metabolic pathways, particularly pyruvate metabolism, the citrate cycle, and ribosome biogenesis (Fig 10).

### DI provides protection against *T. gondii* infection in mice

**Fig 11:**
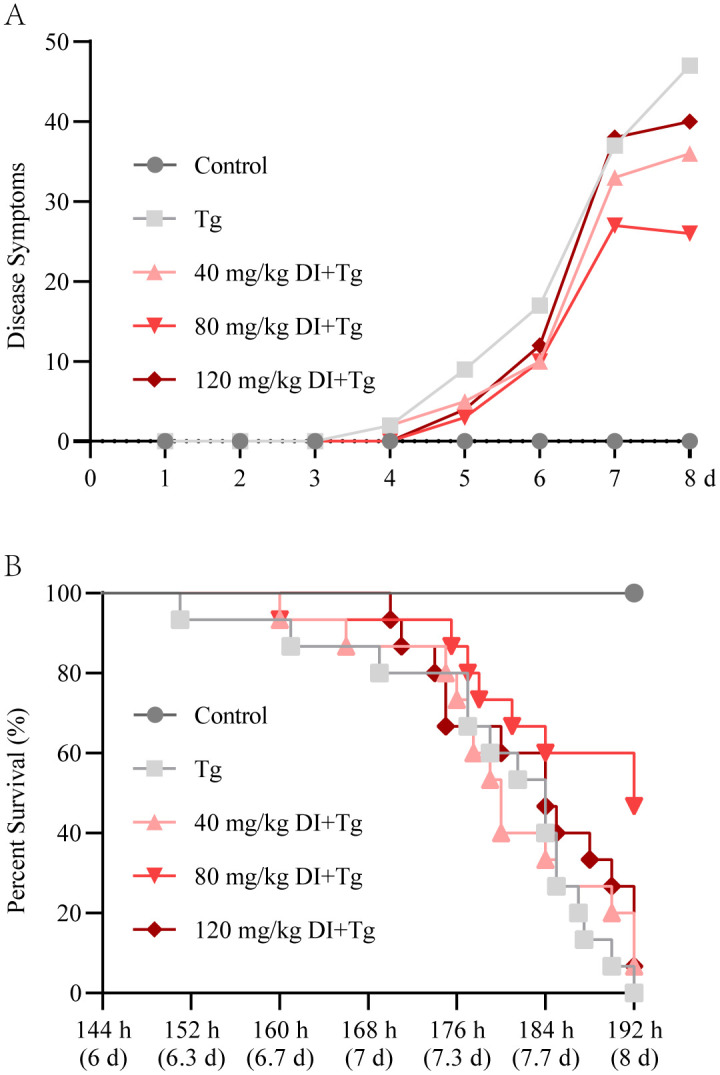
Protective effect of DI in *T. gondii*-infected mice. *T. gondii*-infected mice (Tg group) were intraperitoneally injected with 100 tachyzoites. Four hours post-infection, the DI-treated groups received DI at doses of 40, 80, and 120 mg/kg, while the control group was administered an equivalent volume of a PEG300/ddH_2_O solvent mixture. A: Clinical scores were assessed daily using a standardized cumulative scale. B: Survival rates were monitored over an 8-day observation period.

To validate the protective efficacy of DI against *T. gondii* infection *in vivo*, mice were intraperitoneally challenged with 100 tachyzoites (RH strain) and subsequently treated with DI or a vehicle control starting at 4 hours post-infection. Therapeutic intervention with DI significantly delayed disease progression and alleviated infection-associated clinical symptoms, including piloerection, lethargy, and watery stool (Fig 11A). Notably, DI treatment provided dose-dependent survival benefits, with the 80 mg/kg regimen achieving 46.7% survival at 8 days post-infection, compared to complete mortality in vehicle-treated mice by day 8 (Fig 11B).

### DI inhibits the proliferation of *T. gondii*

**Fig 12:**
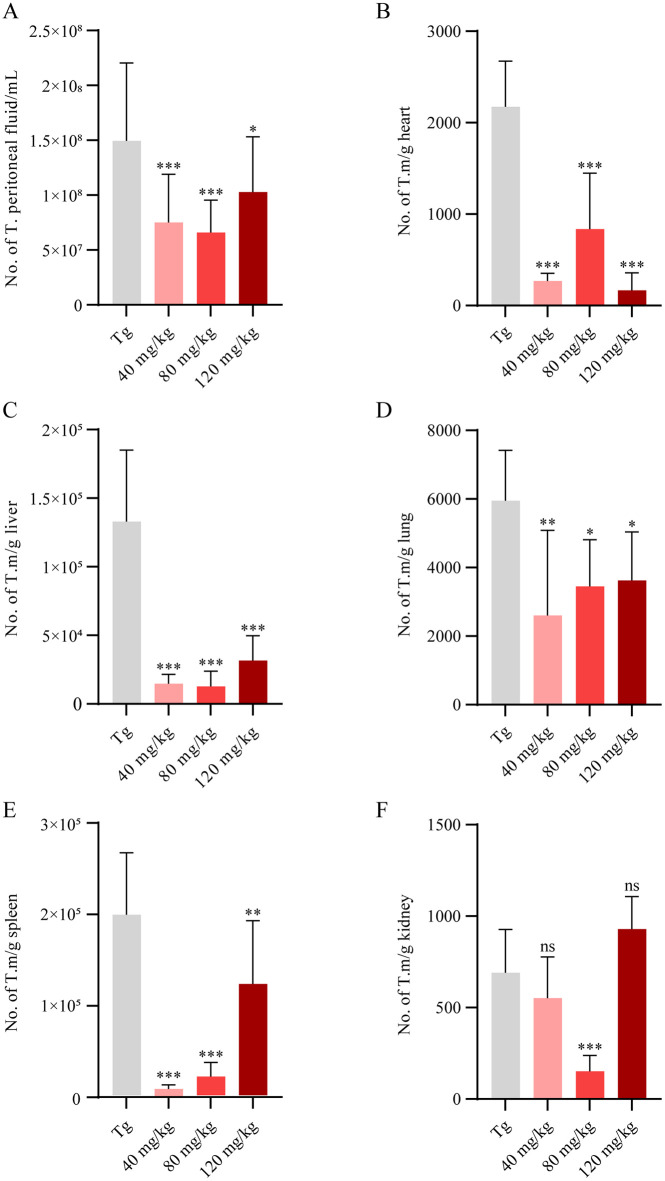
DI treatment reduces *T.gondii* burden in the peritoneal fluid and tissues of infected mice. *T. gondii*-infected mice (Tg group) were intraperitoneally inoculated with 100 tachyzoites. DI-treated groups received DI at doses of 40, 80, or 120 mg/kg, administered four hours post-infection. A Parasite load in mouse peritoneal fluid was determined by direct counting. Data are presented as mean ± SD (n = 15 mice per group). Statistical significance was determined by one-way ANOVA followed by Dunnett’s multiple comparisons test. *p < 0.05, ***p < 0.001. B-F: *T. gondii* DNA levels in cardiac, hepatic, pulmonary, splenic and renal tissues were quantified via qPCR. Data are presented as mean ± SD of 9 biological replicates (mice), with technical triplicates performed for each sample. Statistical significance was determined by one-way ANOVA followed by Dunnett’s multiple comparisons test. *p < 0.05, **p < 0.01, ***p < 0.001, ns, not significant.

The *T. gondii* burden in the heart, liver, lungs, spleen, and kidneys were assessed via qPCR, and tachyzoites in the peritoneal lavage fluid were counted using a hemocytometer. Compared with the control treatment, DI treatment significantly reduced the parasite burden in all tested tissues (Fig 12A-12F), with the most pronounced reduction observed in the liver. Specifically, doses of 40 mg/kg and 80 mg/kg DI markedly decreased parasite load in the peritoneal fluid, liver, heart, spleen, and lungs. However, the efficacy was less evident in the lungs and kidneys. Notably, 80 mg/kg DI demonstrated superior anti-*T. gondii* effects in kidney tissue compared with the other treatment groups.

### DI slightly attenuates liver and spleen lesions in mice infected with *T. gondii*

**Fig 13:**
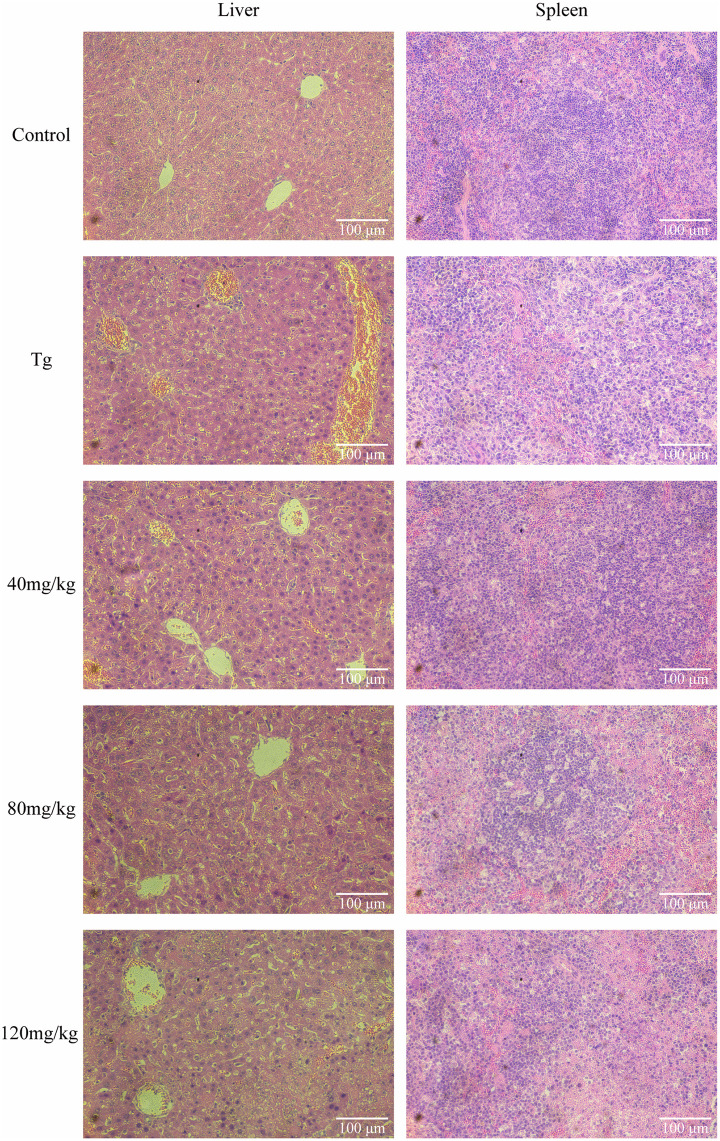
Effects of DI on *T. gondii*-induced histopathological damage in murine hepatic and splenic tissues. (200×).

To further investigate the therapeutic potential of DI in *T. gondii* infection, we analyzed its effects on target tissues. Histopathological evaluation conducted four days post-treatment provided a qualitative assessment of tissue architecture in the liver and spleen. Representative images indicate that DI treatment may attenuate lesion severity, such as hemorrhage, compared to untreated infected controls; however, inflammatory infiltration persisted in the infected mice (Fig 13).

### DI partially reduces tissue damage following *T. gondii* infection in mice

**Fig 14:**
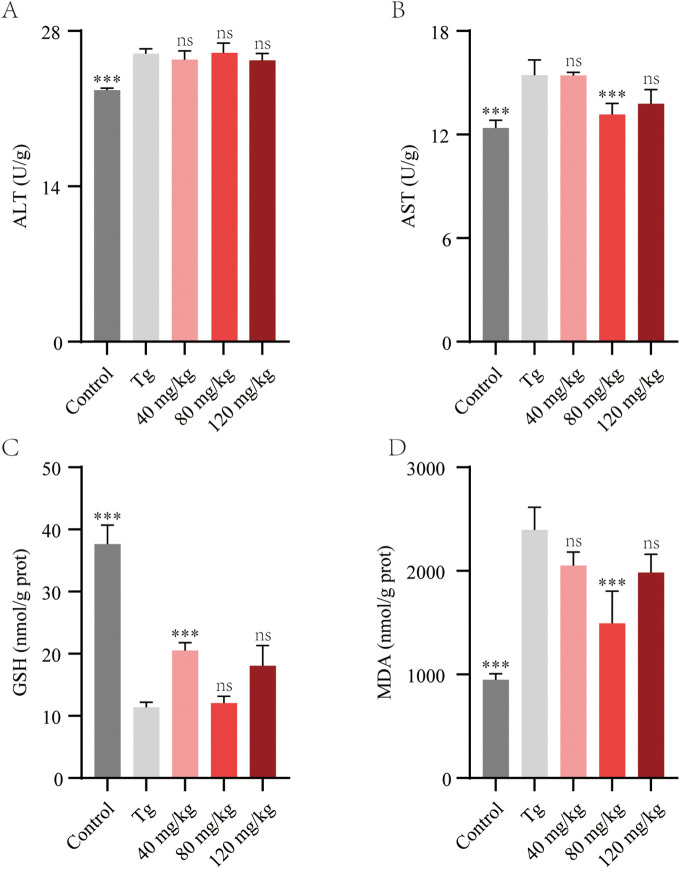
DI ameliorates tissue damage and reduces oxidative stress in *T. gondii*-infected mice. *T. gondii*-infected mice (Tg group) were intraperitoneally inoculated with 100 tachyzoites. DI-treated groups (40, 80, and 120 mg/kg) received DI four hours post-infection. Control group mice were administered equivalent volumes of a PEG300/ddH_2_O solvent mixture. Liver tissues were collected on day 8 post-infection for biochemical analysis. A-D: Levels of ALT, AST, GSH, and MDA in the liver. Data are presented as mean ± SD of 5 biological replicates (mice), with technical triplicates performed for each sample. Statistical significance was determined by one-way ANOVA followed by Dunnett’s multiple comparisons test. ***p < 0.001, ns, not significant.

To systematically evaluate the hepatoprotective efficacy of DI, we measured liver ALT and AST levels as biomarkers of hepatic injury. Biochemical analysis revealed that ALT concentrations did not significantly decrease across the treatment groups (*p* > 0.05, Fig 14A), whereas AST levels were suppressed in a dose-dependent manner, with the 80 mg/kg DI regimen producing a statistically significant reduction compared to the *T. gondii*-infected controls (*p* < 0.001, Fig 14B). These findings suggest that DI provides limited hepatic protection, although its potential therapeutic effects on extrahepatic tissues warrant further investigation.

### DI attenuates oxidative stress following *T. gondii* infection in mice

*T. gondii* infection induces cellular oxidative damage, characterized by elevated levels of MDA, a biomarker of lipid peroxidation. Conversely, GSH, an endogenous low-molecular-weight antioxidant, neutralizes ROS to mitigate infection-associated oxidative stress. Quantitative analysis revealed dose-dependent effects of DI: compared with *T. gondii*-infected controls, treatment with 40 mg/kg DI significantly increased hepatic GSH levels (*p* < 0.001, Fig 14C), whereas 80 mg/kg DI markedly reduced MDA accumulation (*p* < 0.001, Fig 14D). These findings suggest that DI activates endogenous antioxidant defenses to counteract parasite-induced oxidative injury in murine hosts; however, therapeutic optimization requires systematic evaluation of dose-dependent pharmacodynamics.

### DI combats *T. gondii* infection by promoting the release of TNF-α, IFN-γ, and IL-12 from the host

**Fig 15:**
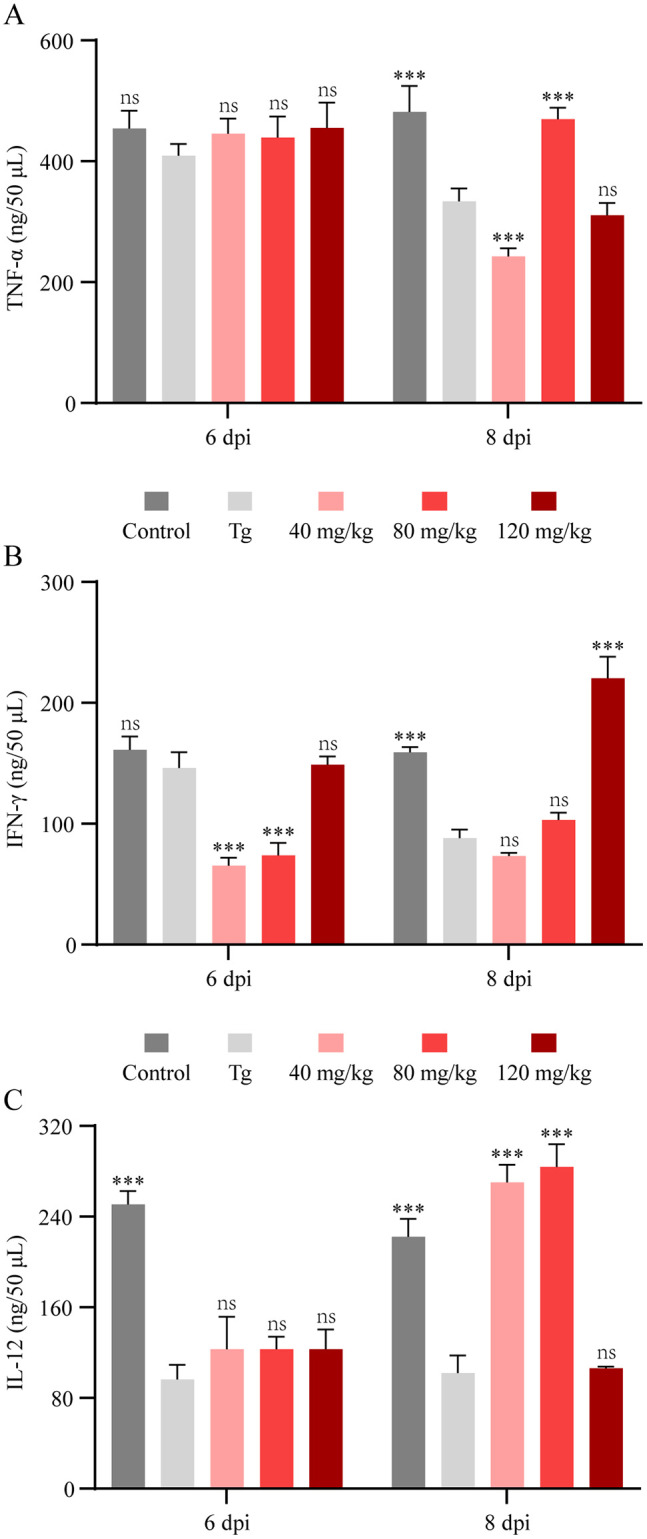
Effect of DI on cytokine levels in mouse serum. *T. gondii*-infected mice (Tg group) were intraperitoneally inoculated with 100 tachyzoites. DI-treated groups (40, 80, and 120 mg/kg) received DI 4 hours post-infection. Control group mice were administered equivalent volumes of a PEG300/ddH_2_O solvent mixture. Blood samples were collected from each group on days 6 and 8 post-infection, and serum was subsequently separated. A-C: Levels of TNF-α, IFN-γ, and IL-12 in serum were measured by ELISA. Data are presented as mean ± SD of 5 biological replicates (mice), with technical triplicates performed for each sample. Statistical significance was determined by two-way ANOVA followed by Dunnett’s multiple comparisons test. ***p < 0.001, ns, not significant.

To investigate the therapeutic effects of DI on *T. gondii*-infected murine models, we systematically quantified serum concentrations of key inflammatory cytokines (TNF-α, IFN-γ, and IL-12) at different stages of infection. The results revealed distinct dose- and time-dependent patterns. TNF-α dynamics (Fig 15A): At 8 days post-infection (dpi), the 40 mg/kg DI group exhibited significantly reduced TNF-α levels compared to the *T. gondii* control group (*p* < 0.05). In contrast, the 80 mg/kg DI group showed a paradoxical upregulation of TNF-α (*p* < 0.001 vs. the Tg group). No significant therapeutic effect was observed with the 120 mg/kg DI treatment. IFN-γ modulation (Fig 15B): Early-phase analysis (6 dpi) revealed potent IFN-γ suppression in both the 40 mg/kg and 80 mg/kg DI groups (*p* < 0.001 vs. the Tg group), whereas the 120 mg/kg DI dose remained ineffective. However, this pattern reversed by 8 dpi, with the 120 mg/kg DI group displaying markedly elevated IFN-γ levels surpassing those of the *T. gondii* infection group (*p* < 0.001), while lower doses showed diminished efficacy. IL-12 regulation (Fig 15C): No significant intergroup differences were detected at 6 dpi. Later-phase analysis (8 dpi) revealed dose-dependent increases in IL-12 in both the 40 mg/kg and 80 mg/kg DI groups (*p* < 0.001 vs. the Tg group). Notably, the 120 mg/kg DI group failed to demonstrate significant IL-12 modulation at either time point.

## Discussion

Itaconic acid, a key immunometabolite produced via immune-responsive gene 1 (*IRG1*)-mediated decarboxylation of cis-aconitate during macrophage activation, exhibits broad-spectrum antimicrobial activity along with anti-inflammatory and antioxidant properties [23]. In contrast to the parent compound, dimethyl itaconate (DI), a structurally modified derivative, has recently attracted significant attention in infectious disease research due to its enhanced membrane permeability and distinct immunomodulatory effects [12]. Despite these advances, the specific antiparasitic activity of DI against *T. gondii* tachyzoites remains poorly understood. Our study presents the first systematic evaluation of DI’s anti-tachyzoite activity of DI using integrated *in vitro* and *in vivo* approaches, including tachyzoite proliferation assays and murine models of acute toxoplasmosis. Mechanistic investigations revealed that DI exerts stage-specific therapeutic effects through a biphasic modulatory mechanism, as demonstrated by multi-omics analyses and functional validation experiments.

The cytotoxicity and safety of DI in host cells are critical for its translational potential. In the present study, the CC₅₀ of DI in BHK-21 cells was determined to be 105.4 μg/mL. All concentrations used in anti-Toxoplasma assays were well below this threshold, confirming that the observed inhibitory effects resulted from direct against *T. gondii* rather than non-specific host cell toxicity. Consistent with previous studies, DI exhibited minimal cytotoxicity in various mammalian cell, including keratinocytes, CD8 ⁺ T cells, and dendritic cells [24,25]. The favorable safety and high selectivity support DI as a promising candidate for treating toxoplasmosis.

*In vitro* experiments revealed that the safe concentration of DI for BHK-21 cells was 73 μg/mL*.* To investigate the underlying mechanism, preliminary transmission electron microscopy (TEM) analyses were conducted, revealing ultrastructural alterations in *T. gondii* tachyzoites induced by DI. These changes included organelle swelling, structural disorganization, and loss of recognizable mitochondrial architecture, along with signs of autophagy activation within the parasites. However, owing to technical limitations during sample preparation and TEM imaging, DI-treated *T. gondii* tachyzoites were unevenly distributed and some were severely disrupted. Accordingly, quantitative analysis of the number of parasites and the proportion of autophagosome-like structures was not performed in this study. Plaque assays and qPCR further demonstrated a concentration-dependent therapeutic effect: higher DI concentrations correlated with reduced plaque formation areas and significantly inhibited tachyzoite proliferation. Collectively, these findings indicate that DI not only protects host cells from *T. gondii* tachyzoite invasion but also suppresses parasite replication in a dose-dependent manner. Nevertheless, the precise developmental stage of *T. gondii* targeted by DI—namely invasion, intracellular proliferation, or egress— remains to be further elucidated. Given the monomorphic mitochondrial structure of *T. gondii* and the critical role of organelles in parasitic survival [26], we systematically examined mitochondrial dysfunction as a potential therapeutic target. Mechanistic validation through mitochondrial membrane potential assays, intracellular ATP quantification, and reactive oxygen species (ROS) detection revealed that DI treatment significantly depolarized mitochondrial membranes, depleted ATP levels, and increased ROS production in tachyzoites. This triad of mitochondrial disruptions demonstrates that DI exerts its anti-*T. gondii* effects primarily through mitochondrial destabilization.

Host-derived cytokines play pivotal roles in counteracting *T. gondii* tachyzoite infections. As the master regulator of Th1-type immune responses, IL-12 orchestrates antiparasitic defenses through two key mechanisms: activating natural killer (NK) cells and CD8 + T lymphocytes to increase IFN-γ production [27], and inducing IFN-γ-mediated upregulation of indoleamine 2,3-dioxygenase (IDO) in host cells [28]. This enzymatic pathway degrades tryptophan, thereby establishing a metabolic barrier that restricts parasitic proliferation. Furthermore, TNF-α directly inhibits *T. gondii* replication in THP-1 cells and synergistically enhances the parasiticidal activity of IFN-γ through nitric oxide (NO)-dependent mechanisms [29–31]. ELISAs revealed distinct cytokine dynamics during infection, with sharp increases in IL-12, IFN-γ, and TNF-α, followed by DI-induced normalization of IL-12 and IFN-γ and sustained TNF-α elevation in *T. gondii*-challenged cells. This cytokine recognition mechanism demonstrates that DI selectively inhibits parasitic infection-induced hyperactivation of the IL-12/IFN-γ signaling axis while preserving TNF-α-mediated pathways, consistent with findings reported by HE [32] and Swain [33].

Transcriptomic analysis revealed critical metabolic perturbations induced by DI treatment. GO enrichment analysis showed significant enrichment in biological processes related to cellular nitrogen compound metabolism and intracellular organelle organization. KEGG pathway analysis further identified pyruvate metabolism and the citrate (TCA) cycle as core pathways involving differentially expressed genes (DEGs), suggesting that modulation of mitochondrial function is a key mechanism underlying altered cellular energy supply and metabolic homeostasis. These findings were corroborated by subsequent assays related to energy metabolism. Pyruvate, the product of glycolysis, requires transport across the mitochondrial membrane and subsequent oxidation within the mitochondria to support aerobic respiration. Our data demonstrated significant upregulation of pyruvate carboxylase (PC) and pyruvate kinase (PK) in DI-treated parasites, suggesting that these enzymatic changes may serve as compensatory mechanisms to counteract disrupted pyruvate homeostasis. *T. gondii* utilizes pyruvate as the central node of carbon metabolism. In this pathway, PC converts pyruvate to oxaloacetate, thereby sustaining substrate provision for the TCA cycle. Moreover, PK directly regulates pyruvate production through glycolysis. Pharmacological inhibition of PK disrupts pyruvate flux, causing cellular energy depletion that ultimately induces parasite death [34]. Intriguingly, we observed significant upregulation of PK expression in treated parasites, suggesting that DI may exert its antiparasitic effects by indirectly impairing PK activity, ultimately disrupting pyruvate metabolic homeostasis. Furthermore, TCA cycle DEG profiling revealed a bifurcated regulatory pattern: upstream enzymatic components (ACLY, MDH) were markedly upregulated, while downstream elements (CS, SDH) exhibited coordinated downregulation. This paradoxical observation indicates that DI induces mitochondrial interference in a phased manner. Taken together, transcriptomic profiling and energy metabolism assays confirm that pyruvate metabolism and the TCA cycle are closely linked to DI treatment. Further studies combining targeted metabolomics and enzyme activity assays are warranted to verify these pathways and construct a more comprehensive mechanistic network.

Mechanistic investigations have uncovered distinct regulatory impacts on mitochondrial energy production: increased ACLY expression enhances acetyl-CoA generation to support lipid metabolism [35], while suppression of citrate synthase (CS) impairs TCA cycle function [36]. This disruption causes α-ketoglutarate to accumulate, which inhibits NADH transfer to the electron transport chain (ETC) through substrate-level inhibition [37]. Energy metabolism analyses showed that DI-induced mitochondrial dysfunction occurs via two combined mechanisms: (1) targeted inhibition of complex II (NDH-2) and complex III (cytochrome bc1), and (2) subsequent cessation of electron transfer. These pathological effects were quantitatively confirmed by marked ATP depletion and total loss of membrane potential, demonstrating a direct causal link between ETC blockade and bioenergetic failure. In summary, this study clarifies how DI exerts anti-Toxoplasma activity by disrupting the pyruvate-TCA-ETC metabolic pathway in a coordinated way, resulting in mitochondrial energy collapse.

*In vivo* investigations have demonstrated that DI provides dose-dependent protection against *T. gondii* infection in murine models. Administration of DI at a dosage of 80 mg/kg markedly reduced clinical symptoms and extended survival duration, indicating the therapeutic potential of this treatment regimen. The study utilized the highly virulent RH strain (Type I) of *T. gondii*, wherein even a minimal inoculum of a single tachyzoite resulted in a 100% mortality rate within eight days post-infection [38]. While this acute infection model substantiates the therapeutic efficacy of DI, the extreme virulence of the parasite constrains the observation period, thereby potentially limiting a thorough assessment of the long-term benefits of the treatment. Additionally, in the present study, DI was administered solely *via* intraperitoneal injection. Although this route provides stable and consistent drug exposure and is appropriate for proof-of-concept investigations of efficacy and mechanism in acute mouse models, it is not suitable for clinical translation. Future studies will therefore evaluate oral administration (e.g., intragastric gavage) to characterize DI’s oral bioavailability, pharmacokinetic properties, and *in vivo* antiparasitic activity, with the aim of improving its clinical applicability as a candidate therapeutic for toxoplasmosis.

Besides survival analysis, a quantitative evaluation of parasitic load was conducted to assess the anti-proliferative effectiveness of DI against *T. gondii* tachyzoites *in vivo*. The number of tachyzoites in both the peritoneal lavage fluid and visceral organs (heart, liver, and spleen) was significantly reduced in the treatment groups compared to the control groups, confirming DI’s systemic inhibitory impact on parasite growth. However, a weaker therapeutic effect was observed in lung and kidney tissues, possibly due to organ-specific immune microenvironments. Interestingly, although the liver showed the greatest reduction in parasite burden, histological examination revealed ongoing inflammatory infiltration and limited tissue repair. This paradox may result from two factors: (1) the brief treatment period (4 days), constrained by the acute virulence of the RH strain, was too short to allow tissue regeneration despite effective parasite suppression; (2) DI’s partial therapeutic effect—while it inhibited tachyzoite replication, it did not completely clear the parasites or physically remove residual ones. These surviving pathogens might still trigger antigenic responses. In addition, our *in vivo* efficacy study did not include a positive control group using effective anti-Toxoplasma drugs such as sulfadiazine or pyrimethamine. The primary objective of the present work was to determine whether DI possesses intrinsic therapeutic activity against *T. gondii in vivo*, rather than to directly compare its potency with clinically approved agents. Accordingly, although our findings demonstrate the antiparasitic potential of DI in animal models, future studies with appropriate positive controls will be necessary to evaluate its relative efficacy and translational promise in comparison with first-line treatments.

Although DI effectively inhibits *T. gondii* proliferation *in vivo*, the invasive nature of tachyzoites inevitably causes irreversible histopathological damage during host invasion, leading to inflammatory responses and hemorrhagic lesions. Therefore, beyond assessing DI’s antiparasitic effectiveness, this study also explored its potential to alleviate tissue damage caused by the parasite. Importantly, DI did not significantly decrease ALT levels in infected mice, indicating limited liver-protective effects. This highlights the necessity for additional hepatoprotective treatments to ensure full liver preservation. Conversely, at a dose of 80 mg/kg, DI significantly reduced systemic AST levels, suggesting it offers partial protection against cellular damage outside the liver in key organs such as the heart, kidneys, and skeletal muscles.

Oxidative stress caused by *T. gondii* leads to considerable pathological damage in infected hosts. Malondialdehyde (MDA) and reduced glutathione (GSH) are important biomarkers used to assess the extent of oxidative stress and the ability of cells to repair damage [39]. Increased MDA levels along with decreased GSH indicate heightened lipid peroxidation and weakened antioxidant defenses [31], while normalization of these markers signifies the recovery of oxidative balance. Quantitative analysis showed that DI influenced redox balance in a dose-dependent manner. Administering 40 mg/kg of DI did not significantly raise MDA levels but did significantly increase hepatic GSH levels, suggesting insufficient antioxidant response to fully counter lipid peroxidation. Treatment with 80 mg/kg DI significantly lowered MDA levels to nearly normal, without altering GSH levels. This dose-dependent effect implies that higher DI doses may activate other antioxidant pathways that help protect tissues.

IL-12, IFN-γ, and TNF-α are pivotal cytokines governing host defense against *T. gondii* tachyzoite infection. Following murine infection, IL-12 drives Th1 cell differentiation through STAT signaling and potently induces IFN-γ and TNF-α production [40–42]. In the present study, infected mice exhibited markedly reduced IL-12 levels at 6- and 8-days post-infection, consistent with the immunosuppressive state characteristic of late-stage infection. Mechanistically, early IL-12 secretion via TLR-MyD88 signaling activates NK cells and dendritic cells to initiate IFN-γ production [43,44], whereas late-phase suppression is associated with parasite-mediated inhibition of the NLRP3 inflammasome and perturbed host metabolism [45]. Itaconate and its derivatives are known to modulate inflammation and metabolism by inhibiting succinate dehydrogenase (SDH) and regulating immunometabolic networks [10,46], and DI can further suppress inflammation *via* the Nrf2 pathway [47]. DI exerts biphasic, dose‑dependent immunomodulatory effects on TNF‑α, IFN‑γ, and IL‑12 that are mechanistically coupled to its direct anti‑parasitic activity *via* mitochondrial metabolic disruption. *In vitro*, DI selectively mitigates infection-driven overproduction of IFN-γ and IL-12 without altering TNF-α, thereby restraining excessive inflammation while preserving core anti-parasitic signaling. *In vivo*, the temporal and dose-dependent cytokine shifts correlate closely with diminished tissue parasite burdens, alleviated oxidative stress (lower MDA, preserved GSH), partially reduced organ damage, and enhanced host survival. The biphasic regulation of IFN-γ and TNF-α is not an isolated immune event but is functionally linked to DI-induced mitochondrial dysfunction in *T. gondii*, including membrane depolarization, ATP depletion, and ROS accumulation, which directly suppress tachyzoite proliferation; early attenuation of pro-inflammatory cytokines relieves immunopathology, while subsequent restoration of IFN-γ and IL-12 strengthens host immune surveillance. Collectively, these results identify DI as a biphasic immunomodulator that regulates protective immunity through distinct temporal mechanisms: early suppression of excessive inflammation followed by late restoration of immune surveillance, which acts in concert with its direct anti-parasitic metabolic effects to control *T. gondii* infection.

## Conclusions

In summary, our research demonstrated that DI effectively suppresses the proliferation of *T. gondii* tachyzoites *in vitro* and shows low cytotoxicity to host cells at antiparasitic concentrations, indicating good selectivity. Transcriptomic analysis revealed that this dual effect is mediated by interference with mitochondrial energy production through alterations in pyruvate metabolism and the TCA cycle. These mechanisms were validated by functional assays targeting mitochondrial energy pathways. Furthermore, DI exhibited potent anti-*T. gondii* activity *in vivo*, enhancing survival rates, reducing parasite burdens in internal organs, and partial attenuating tissue damage. In conclusion, we identified DI as a dual-action anti-Toxoplasma agent with complementary effects both *in vitro* and *in vivo*, highlighting its potential as a therapeutic option for acute toxoplasmosis.

## Supporting information

S1 Data Excel spreadsheet containing, in separate sheets, all numerical values for Figs 1A, 1B, 3F, 4, 5, 7, 8A-8C, 9, 10, 11A, 11B, 12A, 12B-12F, 14 and 15. (XLS)
